# Adaptive Low-Carbon Bioethanol Energy Management through Knowledge Graph–Digital Twin Frameworks

**DOI:** 10.1021/acsomega.6c06697

**Published:** 2026-09-23

**Authors:** Yitong Niu, Ying Ying Tye, Chee Keong Lee, Cheu Peng Leh

**Affiliations:** School of Industrial Technology, 26689 Universiti Sains Malaysia, 11800 minden Heights, Pinang 11800, Malaysia

## Abstract

Lignocellulosic bioethanol can reduce dependence on fossil liquid fuels, but its low-carbon performance is strongly constrained by process-energy demand. Energy use is shaped by coupled variations in feedstock quality, pretreatment response, fermentation performance, separation efficiency, equipment condition, and utility integration. Existing energy-balance, process-simulation, techno-economic, life-cycle, and data-driven studies have provided important methods for quantifying energy use, cost, emissions, and process bottlenecks, but their outputs often remain distributed across separate models and assessment boundaries. This fragmentation limits the ability to explain why energy intensity changes during operation and which intervention should be prioritized. A knowledge graph–digital twin approach is proposed to connect semantic representation with dynamic process interpretation for low-carbon bioethanol energy management. Feedstock, process, equipment, energy, sustainability, and decision entities are organized into traceable relationships, while a literature-derived metric system links process variables, sustainability indicators, equipment-state descriptors, data-readiness metrics, and heat-integration information. Economic feasibility is further considered by relating implementation and life-cycle costs to measurable operational benefits and staged deployment decisions. The framework reframes bioethanol energy analysis from retrospective accounting toward explainable, updatable, and decision-oriented energy management.

## Graphical abstract

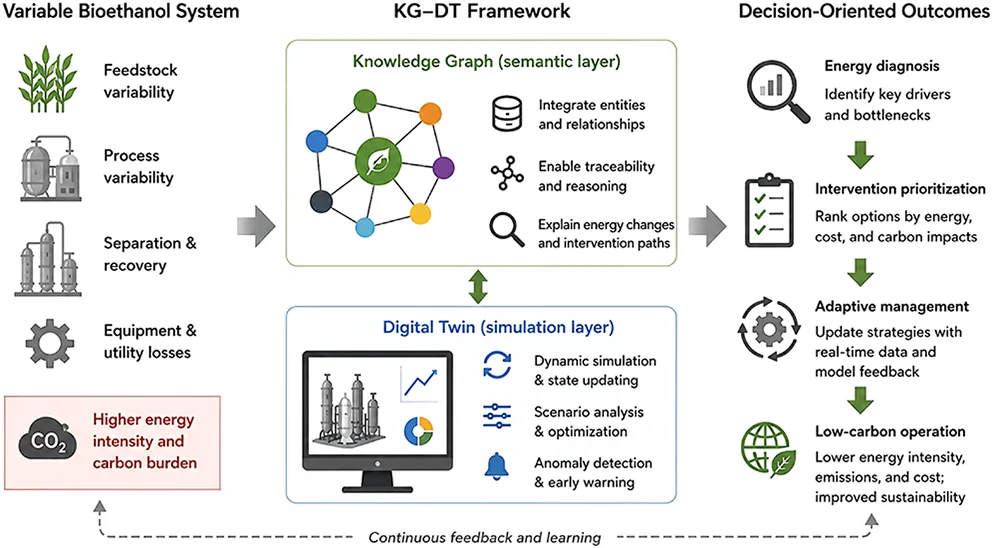

## 1 Introduction

Lignocellulosic bioethanol has been widely examined as a second-generation liquid fuel because it can use nonfood biomass, agricultural residues, forestry residues, and dedicated energy crops rather than starch- or sugar-based feedstocks.^1,2^ Its climate benefit is not inherent to the feedstock, since life-cycle greenhouse gas and energy results vary with feedstock supply, conversion yield, process configuration, energy source, allocation method, coproduct treatment, and system boundary.^3,4^ The biochemical route also requires a more complex process chain than first-generation ethanol, with pretreatment, hydrolysis, fermentation, product recovery, wastewater treatment, and utility generation forming an integrated production system.^5,6^ Within this system, energy intensity is a limiting factor because pretreatment heat demand, ethanol recovery, dehydration, and heat integration strongly influence external steam and electricity requirements.^7–9^ Low-carbon bioethanol should therefore be framed not only as a conversion technology but also as an energy-management problem across feedstock, process, separation, and utility layers.

Energy analysis in bioethanol systems has developed through several complementary modeling traditions. Energy-balance studies established the basic comparison among fossil energy input, renewable fuel output, and net energy performance for ethanol pathways.^10,11^ Process simulation and techno-economic analysis then provided more detailed representations of unit operations, capital cost, operating cost, and sensitivity to conversion assumptions.^8,12^ Life-cycle assessment broadened the evaluation by linking process-energy use with greenhouse gas emissions, fossil energy displacement, coproduct treatment, and regional production contexts.^13,14^ Heat-integration and optimization studies further showed that utility demand can be reduced by redesigning heat-recovery networks, process configurations, and energy supply strategies.^15,16^ More recently, real-time monitoring, soft sensors, and data-driven models have been developed to improve state estimation, control, and optimization in lignocellulosic ethanol fermentation.^17^ These studies provide essential analytical foundations, but they usually remain organized around separate objectives such as energy accounting, process design, environmental assessment, cost estimation, heat integration, or process monitoring.

The main limitation is not that bioethanol lacks energy models, but that the variables driving energy use are usually treated in separate analytical layers. Variations in biomass moisture and particle-size distribution can already disturb feeding and preprocessing before the material reaches the reactor, while differences in composition and recalcitrance change the energy and chemical requirements needed to obtain fermentable sugars.^18–20^ Pretreatment severity and chemistry further shape downstream performance by changing sugar release and generating inhibitory compounds such as furfural, hydroxymethylfurfural, acetic acid, and phenolics.^21,22^ Fermentation performance then determines ethanol titer and residual sugar concentration, which directly affect product-recovery load and the energy required for concentration, distillation, and dehydration.^23^ At the same time, equipment degradation and maintenance-related conditions, including heat-transfer deterioration and fouling, can create energy penalties that are difficult to separate from feedstock- or conversion-driven variation using static assessment alone.^24,25^ As a result, operators may observe higher energy intensity without being able to trace whether the dominant cause lies in upstream feedstock quality, biochemical conversion, downstream separation, or equipment degradation. The missing layer is therefore a semantic–dynamic structure that can connect process variables, equipment states, energy metrics, and intervention options in an interpretable form.

Knowledge graphs and digital twins provide complementary functions for addressing this integration gap. Digital twins can represent physical assets or processes through models that support monitoring, state estimation, simulation, control, and optimization in bioprocess and biomanufacturing systems.^26,27^ Knowledge graphs and ontologies can organize heterogeneous entities, attributes, and relationships into machine-readable semantic structures that support interoperability, querying, and reasoning across complex industrial data environments.^28,29^ Their integration is increasingly discussed as a way to connect data streams, simulation models, domain rules, and decision logic within digital-twin architectures.^30,31^ In bioethanol energy management, this combination can link feedstock properties, unit operations, equipment states, energy indicators, carbon metrics, and intervention options within a single semantic–dynamic framework. As shown in Scheme 1, this Review frames the transition from static, retrospective energy accounting to an adaptive KG–DT approach in which dynamic process information, semantic reasoning, simulation, and decision feedback are connected.

**Scheme 1:**
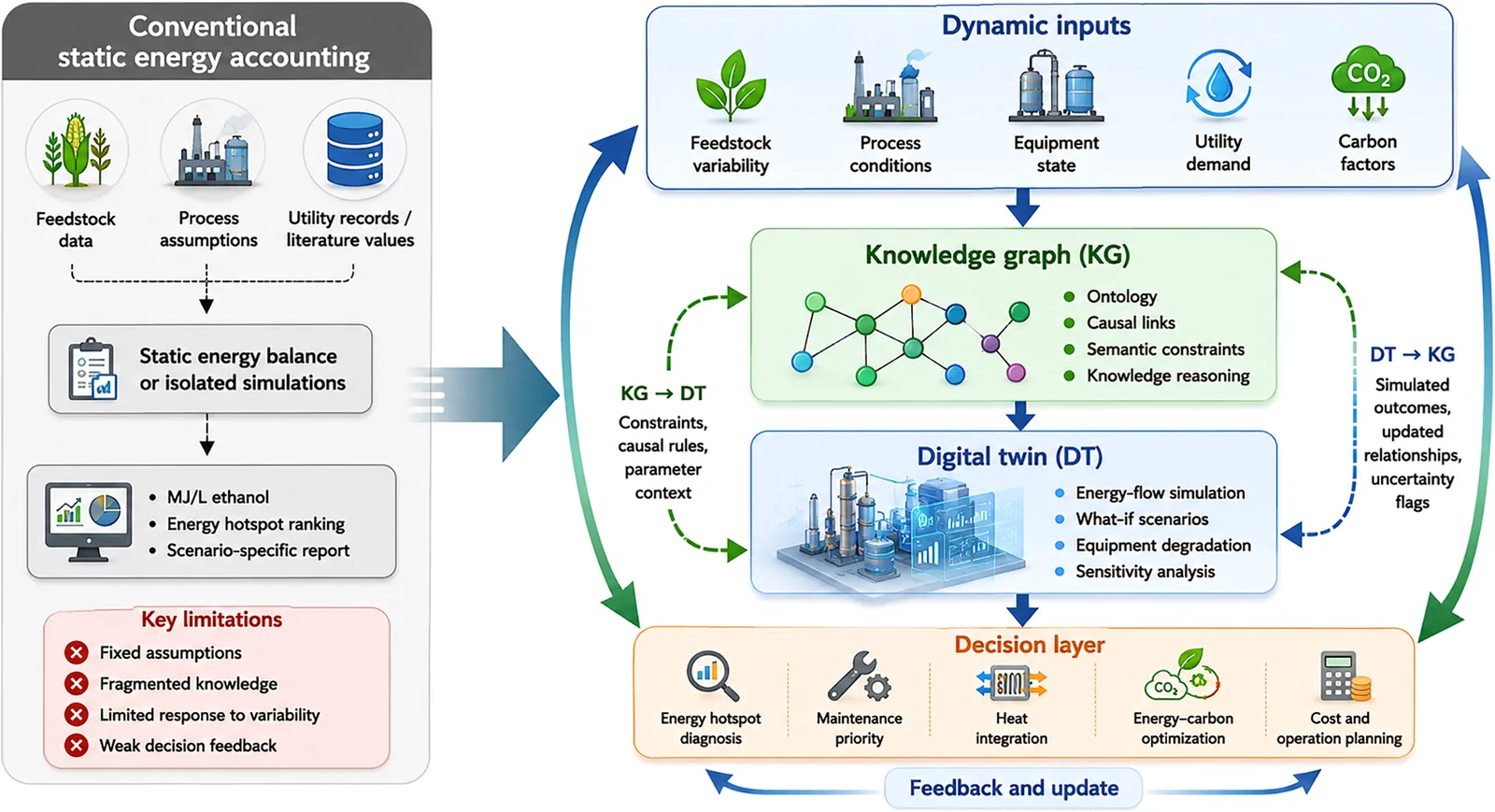
Conceptual Transition from Static Bioethanol Energy Accounting to Adaptive KG–DT Energy Management

A KG–DT framework is therefore useful only if it moves beyond adding another digital layer to an already complex process. Its value lies in reorganizing bioethanol energy analysis around traceable relationships among material variability, conversion performance, equipment condition, utility demand, carbon burden, and operational response. In this review, KG–DT integration is developed as a review-derived framework for adaptive low-carbon bioethanol energy management rather than as a plant-validated control system. The proposed framework links literature-based energy knowledge with semantic representation, dynamic scenario analysis, and decision-oriented interpretation. Its practical feasibility is further examined by relating implementation and life-cycle costs to measurable operational benefits and staged deployment decisions. An illustrative moisture-distillation efficiency case is used to show how coupled disturbances can be translated into intervention priorities, but the case is intended as a conceptual decision map rather than industrial validation. The central argument is that low-carbon bioethanol requires explainable and updatable energy-management systems capable of identifying not only when energy intensity increases but also why the penalty emerges and which intervention should be prioritized.

## 2 Energy Analysis and Dynamic Drivers in Bioethanol Systems

### 2.1 Conventional Energy-Analysis Methods

Energy analysis in bioethanol systems has developed through several complementary methodologies. Early energy-balance studies established the basis for comparing fossil energy inputs, renewable fuel outputs, net energy ratios, and environmental trade-offs across ethanol pathways.^10,11^ Process design and simulation studies then extended this accounting logic by assigning mass and energy flows to unit operations, allowing pretreatment, hydrolysis, fermentation, separation, and utility systems to be evaluated under defined process configurations.^32^ Techno-economic analysis added capital cost, operating cost, feedstock cost, enzyme cost, utility cost, and minimum ethanol selling price as decision-relevant indicators.^12,33^ Life-cycle assessment broadened the system boundary by connecting process-energy demand with greenhouse gas emissions, fossil energy displacement, regional energy supply, and coproduct treatment.^14,34^ Heat-integration, exergy, and optimization studies further showed that energy performance depends not only on unit-level conversion efficiency but also on heat recovery, steam-network design, thermodynamic losses, and process integration.^15,16,35^ More recent monitoring and data-driven studies have introduced soft sensors, process analytics, and optimization tools for lignocellulosic ethanol fermentation and related bioprocesses.^36,37^ Together, these methods provide the analytical foundation for bioethanol energy assessment, but they were developed for different purposes and are rarely integrated into a single decision-oriented structure.

Energy-balance, process-simulation, and techno-economic studies remain the most direct tools for identifying energy and cost drivers in ethanol production. Energy-balance analysis establishes relationships among biomass input, fossil energy use, fuel output, and net energy performance, while process simulation distributes heat, electricity, material flows, and separation loads across unit operations.^38^ Techno-economic analysis then translates these process assumptions into capital cost, operating cost, feedstock cost, enzyme cost, utility cost, and minimum ethanol selling price.^33,39^ Sensitivity studies further show that economic feasibility is strongly affected by yield, enzyme loading, solids concentration, feedstock price, capital intensity, and energy demand.^40,41^ These methods are essential for benchmarking pathways and comparing the design scenarios. However, because they are usually based on steady-state or scenario-specific assumptions, their outputs are not naturally structured to diagnose real-time deviations caused by feedstock variability, process drift, or equipment degradation.

Life cycle, heat integration, and exergy methods extend the analysis from process feasibility to sustainability and thermodynamic efficiency. Life-cycle assessment connects process-energy demand with greenhouse gas emissions, fossil energy displacement, water use, chemical inputs, land-related assumptions, and coproduct allocation.^42–44^ Heat-integration studies show that external steam demand can be reduced through heat recovery, combined heat and power, evaporation integration, and process-network redesign.^45,46^ Exergy analysis further distinguishes energy quantity from energy quality and can identify major thermodynamic losses across lignocellulosic conversion and utility operations.^47,48^ These methods clarify where energy and carbon burdens arise at the system level. They do not, however, directly determine which operational intervention should be prioritized when the same increase in energy intensity can result from different causes such as wet feedstock, low ethanol titer, heat-exchanger fouling, or reduced distillation efficiency.

Digital and data-driven methods have begun to address the operational dimension of bioethanol energy management. Real-time monitoring studies show that off-gas analysis, liquid-phase measurements, biomass tracking, and soft sensors can improve control of lignocellulosic ethanol fermentation.^36^ Machine-learning models have been used to predict bioethanol yield, evaluate pretreatment effects, and support optimization when mechanistic descriptions are incomplete.^49–51^ Digital-twin studies in bioprocessing further suggest that dynamic models can support state estimation, scenario testing, fault detection, and process optimization.^52–54^ These developments move bioethanol analysis toward adaptive operation, but many remain task-specific, such as predicting ethanol concentration, estimating conversion yield, detecting anomalies, or optimizing a single operating window. Without a semantic layer that links feedstock properties, process conditions, equipment states, energy metrics, carbon outcomes, and intervention options, the outputs of energy accounting, TEA, LCA, simulation, monitoring, and optimization remain difficult to translate into traceable operational decisions. Table 1 summarizes representative studies across these methodological streams and highlights the specific gap that motivates KG–DT integration.

**Table 1:** Representative Studies on Energy Analysis, Simulation, Sustainability Assessment, and Digital Optimization Relevant to KG–DT-Based Bioethanol Energy Management

category	system/feedstock	method used	main energy-related focus	key finding	limitation relevant to KG–DT	ref
Energy balance/process-level accounting	Brazilian sugar cane ethanol	Industrial-scale energy and GHG balance	Fossil energy input, plant energy self-sufficiency, GHG balance	Bagasse-based heat and power supply enables favorable energy balance and low fossil energy dependence in sugar cane ethanol systems.	Uses static sector-level averages and does not represent unit-level operating states, equipment degradation, or semantic process relationships.	^55^
	Corn-stover ethanol	Process-chain energy and environmental assessment	Fossil energy displacement, energy return, system-boundary effects	Corn-stover ethanol can reduce fossil energy use and GHG emissions, but results depend strongly on residue removal and boundary assumptions.	Scenario-based and parameter-average assessment; limited ability to connect operating disturbances with equipment-state feedback.	^10^
	U.S. ethanol pathways	Integrated energy, carbon, and ecological footprint assessment	Net energy, carbon balance, petroleum displacement	The energy and carbon performance of ethanol depends strongly on feedstock choice and boundary definition, rather than being inherently low-carbon.	Macro-level comparison without process-control variables, plant-state updating, or causal traceability.	^11^
	Corn-stover lignocellulosic ethanol	NREL mass/energy balance and process economics	Steam demand, electricity demand, utilities, and process baseline	Established a detailed dilute-acid pretreatment–enzymatic hydrolysis–fermentation process baseline widely used in later TEA and LCA studies.	Fixed design-case model; cannot adaptively represent feedstock variability, equipment degradation, or online reasoning.	^5^
	Corn-stover lignocellulosic ethanol	NREL state-of-technology design	Process-energy hotspots, steam/electricity demand, process integration	Updated the biochemical conversion baseline and provided a reference for simulation, TEA, LCA, and sensitivity analysis of 2G ethanol systems.	Offline design benchmark; lacks semantic knowledge representation and real-time feedback between process data and decision making.	^6^
Process simulation and heat integration	Lignocellulosic ethanol	Integrated process-scheme comparison and energy analysis	Process configuration, external energy demand, residue-based energy supply	Process integration and residue-based cogeneration strongly influence external energy requirements in lignocellulosic ethanol production.	Offline scheme comparison; limited synchronization with plant data and no semantic representation of process–energy relationships.	^56^
	First- and second-generation fuel ethanol	Review of process design and integration trends	Process intensification, separation coupling, energy integration	Reaction–separation coupling and energy integration are central opportunities for improving ethanol process efficiency.	Provides process-design insights but not an executable adaptive model or semantic decision layer.	^7^
	Lignocellulosic ethanol	Aspen Plus rigorous process simulation	Whole-process energy demand and utility hotspots	Aspen-based flowsheeting can identify process utility hotspots from pretreatment to ethanol purification.	Mainly an offline simulation; does not include equipment-health feedback, sensor data, or knowledge reasoning.	^32^
	Sugar cane sugar–ethanol production	Process steam-demand reduction and cogeneration assessment	Steam demand and surplus electricity generation	Reducing process steam demand can increase surplus electricity export from sugar cane ethanol plants.	Focuses on thermal-system design but does not integrate feedstock quality, equipment state, and decision rules in a unified framework.	^57^
	Straw-based lignocellulosic ethanol	Pinch analysis and heat-exchanger network design	Heating demand and heat-recovery potential	Heat-exchanger network optimization can substantially reduce external heating demand in lignocellulosic ethanol production.	Based on fixed operating conditions; difficult to absorb real-time disturbances, fouling, or abnormal-event information.	^9^
TEA/LCA	Lignocellulosic ethanol from different feedstocks	Techno-economic analysis	Feedstock effects on cost and process feasibility	Feedstock properties and conversion yields strongly affect ethanol-production cost; pretreatment and feedstock cost are dominant drivers.	Provides static economic outputs but does not map cost drivers to online operating decisions.	^33^
	Lignocellulosic ethanol	Review of techno-economic analysis	Cost drivers and process bottlenecks	Feedstock cost, enzyme loading, capital intensity, conversion yield, and coproduct credits dominate TEA conclusions.	Integrates published scenarios but does not support real-time adaptive energy management.	^12^
	Brazilian sugar cane products	Life-cycle assessment	GHG emissions, energy use, coproduct credits	Brazilian sugar cane products show favorable energy and GHG performance, with coproduct energy credits playing an important role.	Highly dependent on system boundary and average inventory data; lacks real-time plant-state mapping.	^13^
	Corn-stover ethanol in China	Techno-economic analysis	Cost feasibility and parameter sensitivity	Economic feasibility is strongly constrained by feedstock cost and conversion efficiency under the Chinese production context.	Project-level feasibility view rather than adaptive operational optimization.	^58^
	Corn-stover ethanol in China	Life-cycle assessment	Life-cycle energy use and GHG emissions	Future technology scenarios can further improve GHG and energy performance of corn stover ethanol.	Annual or project-level assessment; does not support equipment-level causal tracing or online decision feedback.	^14^
Feedstock and operational variability	Steam-pretreated switchgrass and sugar cane bagasse	Experimental study	Effect of biomass moisture on ethanol yield	Initial biomass moisture content affects ethanol yield after steam pretreatment.	Examines a specific variable without cross-unit semantic linkage or plant-level energy decision integration.	^59^
	Woody biomass pretreatment	Review and energy-consumption evaluation	Pretreatment energy and size-reduction energy	Pretreatment assessment should include energy consumption, not just sugar yield; postchemical size reduction can lower energy demand.	Offline evaluation; does not formalize causal rules into a reusable queryable knowledge layer.	^60^
	Corn-stover and blended biomass feedstocks	Experimental and cost analysis	Feedstock variability, blending, pretreatment response, and cost	Feedstock type, harvesting mode, blending, and pretreatment conditions affect sugar yield and production cost.	Links yield and cost but lacks online state estimation and semantic rule-based reasoning.	^61^
	Corn stover and wheat straw	Experimental, economic, and environmental analysis	Solids-loading effects on yield, economy, and environmental performance	Solids loading changes conversion, economic performance, and environmental impacts; no single condition optimizes all objectives.	Context-specific variable analysis without reusable KG representation.	^62^
	Corn-stover hydrolysate fermentation	Integrated process design and experimental validation	Ethanol titer threshold and distillation feasibility	Integrated design can raise ethanol concentration above the minimum distillation-relevant threshold while maintaining high yield.	Focuses on fermentation–distillation threshold but does not encode such threshold knowledge as explicit queryable rules.	^63^
Digital twin/Industry 4.0	Biomanufacturing, including 2G ethanol fermentation case	Digital-twin framework and fermentation case	Operational efficiency and resource utilization; explicit energy metrics not reported	Proposed a pathway toward full-function digital twins and demonstrated the concept in a bench-scale 2G ethanol fermentation case.	No KG-based semantic layer for cross-unit or cross-asset knowledge reuse.	^64^
	General bioprocesses	Mechanistic digital-twin and control-strategy development	Monitoring, control, and optimization; explicit energy metrics not reported	Digital twins can represent biological, physicochemical, equipment, and peripheral systems to support control and automation.	Emphasizes modeling and control but has limited semantic interoperability and knowledge-reasoning capability.	^27^
	Yeast cultivation and enzymatic hydrolysis	Modular mechanistic digital-twin core-model assembly	Efficient model development; explicit energy metrics not reported	Automated submodel assembly can reduce digital-twin development and computation time substantially.	Lacks KG-style entity-relationship representation and plant-level energy semantics.	^52^
	Smart manufacturing process systems	Integrated KG–DT framework	Semantic interoperability and autonomous optimization; explicit energy metrics not reported	KG–DT integration can improve autonomy, flexibility, and semantic organization in manufacturing workflows.	Demonstrated mainly in manufacturing rather than bioethanol energy systems.	^30^
	General upstream and downstream bioprocesses	Review of digital-twin applications	Monitoring, control, and optimization potential; explicit energy metrics not reported	Digital twins are promising for bioprocess monitoring and optimization, but practical barriers remain in sensing, data, modeling, and validation.	Identifies barriers but does not propose a bioethanol-specific KG-based semantic solution.	^65^
AI/ML and data-driven optimization	Sugar cane bagasse lignocellulosic ethanol by SHF	ANN, C5.0, and random forest	Effects of operating variables; explicit energy metrics not reported	ML models can learn nonlinear effects of temperature, enzyme loading, biomass loading, inoculum, and time on ethanol production.	Single-task prediction without mechanistic constraints or bidirectional synchronization with a DT.	^66^
	Ionic-liquid-pretreated lignocellulosic ethanol	ANN and random forest	Ethanol yield prediction; explicit energy metrics not reported	ML can rapidly predict ethanol yield for different pretreatment–biomass combinations and reduce trial-and-error experimentation.	Limited interpretability, transferability, and real-time capability; not coupled with DT or a knowledge layer.	^67^
	Herbaceous and corn-stover lignocellulosic ethanol	ML, optimization algorithm, and GREET1	Ethanol yield, NaOH consumption, and GHG emissions	ML–LCA integration can improve yield prediction and identify operating conditions that reduce GHG emissions.	Data-driven and offline; lacks online semantic reasoning and plant-state synchronization.	^50^
	Intensified sugar cane ethanol fermentation	Deep-learning soft sensor and PAT	Real-time state estimation; explicit energy metrics not reported	Deep-learning soft sensors can estimate ethanol and substrate concentrations in real time for intensified fermentation monitoring.	Local variable prediction; not extended to plant-level energy decision loops.	^68^
	Corn-stover SSCF ethanol	XGBoost/DNN and genetic algorithm	Yield optimization and parameter importance; explicit energy metrics not reported	XGBoost/DNN models combined with genetic algorithms can rapidly identify optimized operating conditions for ethanol production.	Optimization targets yield rather than integrated energy, equipment state, and knowledge-constrained closed-loop decisions.	^69^

### 2.2 Dynamic Sources of Energy Variability

Energy demand in lignocellulosic ethanol production is not a fixed property of a process route. It changes with feedstock quality, conversion performance, equipment conditions, and utility system behavior. Variations in biomass moisture and particle-size distribution can already disturb feeding and preprocessing before the material reaches the reactor, while differences in composition and recalcitrance change the energy and chemical requirements needed to obtain fermentable sugars.^18,70^ Downstream energy demand is likewise coupled to conversion performance: pretreatment-derived inhibitors can impair fermentation, while low ethanol titer increases the burden of recovery, distillation, and dehydration.^71,72^ Comparable fermentation-based valorization studies of agricultural residues have also evaluated process configuration in terms of recoverable energy, showing that biological conversion performance can be interpreted within a broader energy-recovery context.^73^ The operational problem is therefore diagnostic as well as quantitative: an increase in the total energy demand may reflect upstream feedstock variation, a biochemical conversion bottleneck, downstream separation loss, or utility inefficiency. Static energy indicators can identify the penalty but do not necessarily identify its origin or the appropriate intervention.

Feedstock variability is the first source of uncertainty because lignocellulosic biomass is heterogeneous by origin, harvest conditions, storage history, and preprocessing route. Moisture affects the heating demand, transport efficiency, biological degradation during storage, and consistency of feeding into downstream reactors. Particle size and bulk density influence the milling energy, conveying stability, slurry handling, and solids loading. Compositional variation in cellulose, hemicellulose, lignin, extractives, and ash changes theoretical sugar availability and pretreatment response, while inorganic species can contribute to fouling, corrosion, neutralization demand, or ash-related handling problems.^74–76^ These properties are often measured as feedstock descriptors, but in an energy-management context, they are better treated as upstream disturbances that propagate into pretreatment severity, hydrolysis yield, fermentation robustness, and separation load.

Process variability further amplifies the upstream differences. Pretreatment temperature, residence time, acid or alkali loading, liquid-to-solid ratio, and severity determine solid recovery, sugar release, delignification, hemicellulose solubilization, and inhibitor formation.^77,78^ More severe pretreatment can improve digestibility, but it can also increase chemical demand, reduce fermentable sugar recovery, and form furfural, hydroxymethylfurfural, acetic acid, and phenolic compounds that impair fermentation.^79,80^ Enzymatic hydrolysis then introduces additional coupling among solid loading, enzyme dosage, mixing demand, viscosity, glucose release, and process time.^81,82^ Fermentation performance is especially important for energy management because ethanol titer determines the dilution of the product stream entering separation; low titers increase the energy required for concentration, distillation, and dehydration.^83–86^ Thus, the conversion yield and downstream energy cannot be optimized independently. A condition that improves one unit operation may still increase plant-level energy intensity if it lowers titer, increases inhibitors, extends the residence time, or shifts energy demand to separation.

Equipment and utility systems add a second dynamic layer that is not captured by feedstock or conversion models alone. Distillation columns, molecular-sieve dehydration, reboilers, condensers, heat exchangers, boilers, pumps, and cooling systems determine how biochemical performance is translated into final energy demand. Ethanol recovery is particularly energy-sensitive because dilute fermentation broths require large heat inputs for concentration before dehydration.^87,88^ Heat-exchanger fouling reduces heat-transfer performance and can increase steam consumption, cooling demand, pumping work, and cleaning frequency, while boiler efficiency and steam-network losses affect the external energy required to satisfy the same process load.^89,90^ Equipment degradation can therefore make two operating periods with similar feedstock compositions and ethanol titer showing different energy intensities. As shown in [Fig fig1], energy demand in lignocellulosic bioethanol is shaped by coupled upstream, conversion, separation, equipment, and utility drivers rather than by isolated unit operations. This coupling explains why adaptive energy management requires more than energy accounting: the same rise in energy intensity may require feedstock blending, pretreatment adjustment, fermentation diagnosis, column maintenance, or heat-integration redesign depending on the source of the disturbance.

**Figure 1:**
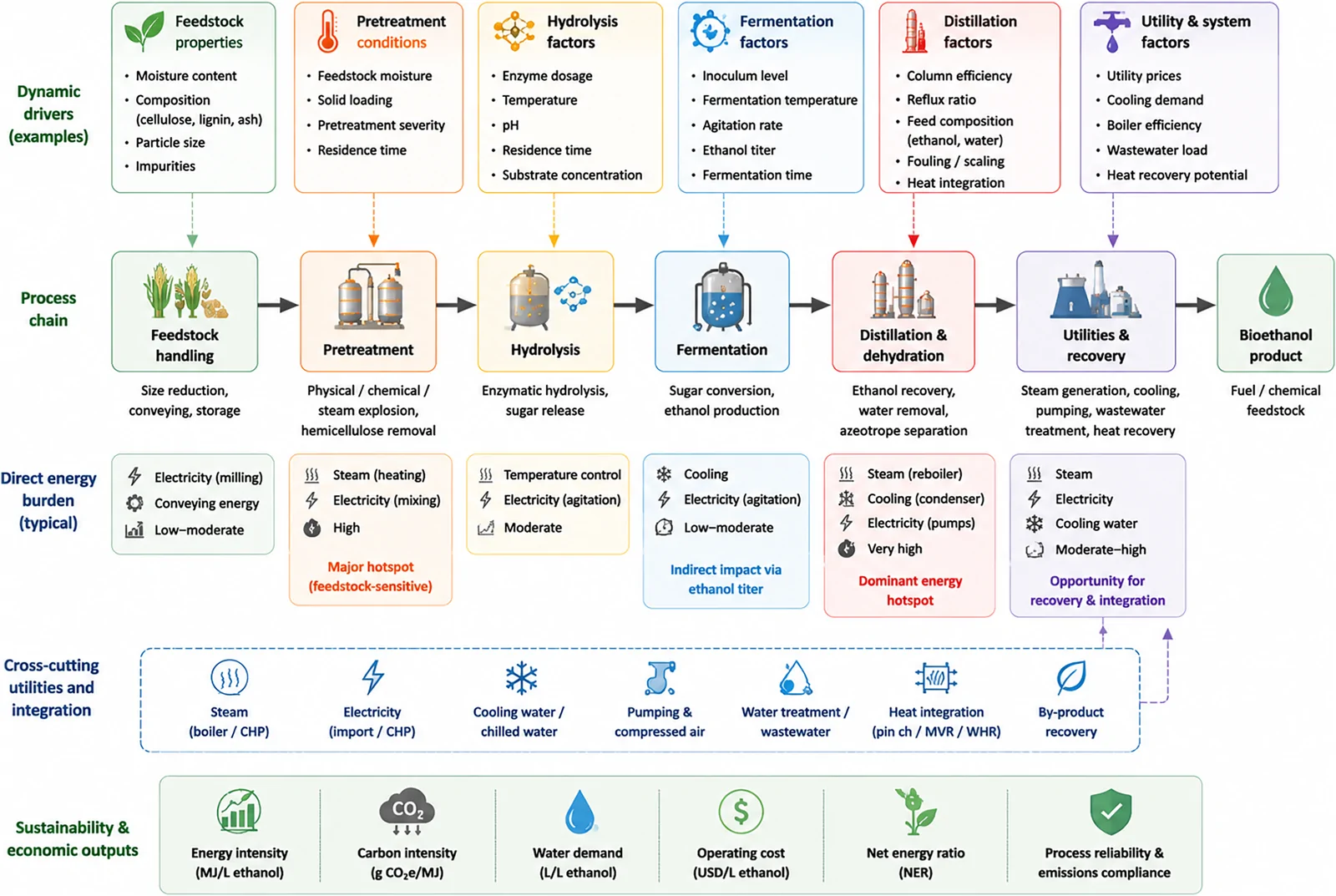
Energy hotspots and dynamic drivers in lignocellulosic bioethanol production.

## 3 KG–DT Framework for Adaptive Energy Management

### 3.1 Semantic Structure and Causal Reasoning

The proposed KG–DT framework begins with semantic organization rather than simulation. A knowledge graph represents entities and relationships in a graph-based structure, and ontologies can provide the schema layer needed for consistent naming, relationship definition, interoperability, and reasoning across heterogeneous data sources.^91,92^ This capability is important for bioethanol energy management because relevant variables are distributed across different domains, including feedstock analysis, process operation, equipment monitoring, utility accounting, sustainability assessment, and decision records. Without a semantic structure, these variables remain as separate data tables or model inputs; with a knowledge graph, they can be represented as connected entities whose relationships are explicit and queryable.

In the proposed ontology, the bioethanol system is organized into six connected layers: feedstock, process, equipment, energy, sustainability, and decisions. The feedstock layer describes material properties such as moisture, carbohydrate composition, lignin, ash, particle size, and storage conditions. The process layer represents pretreatment, hydrolysis, fermentation, distillation, dehydration, and utilities operations. The equipment layer links reactors, fermenters, heat exchangers, boilers, pumps, and columns to condition indicators such as heat-transfer performance, efficiency loss, fouling, vibration, and maintenance state. The energy and sustainability layers connect unit-level heat and electricity demand to plant-level energy intensity, carbon intensity, water use, operating cost, and other performance indicators. The decision layer represents possible interventions, such as feedstock blending, moisture control, pretreatment adjustment, enzyme dosing changes, column maintenance, heat-recovery improvement, and utility optimization.

As shown in Figure 2, the value of the ontology is not only classification, but causal traceability. A feedstock-related query may connect high moisture to an increased pretreatment heat demand, altered solids loading, lower ethanol concentration, and greater separation duty. An equipment-related query may connect reduced column efficiency to higher reboiler duty, increased carbon intensity, and a maintenance priority. These relationships allow the same observed energy penalty to be interpreted through different causal routes. For example, a rise in the total energy demand may be traced to wet feedstock, lower hydrolysis conversion, low fermentation titer, heat-exchanger fouling, or declining distillation performance. A semantic structure therefore provides the diagnostic layer needed before dynamic simulation and optimization can produce actionable recommendations.

**Figure 2:**
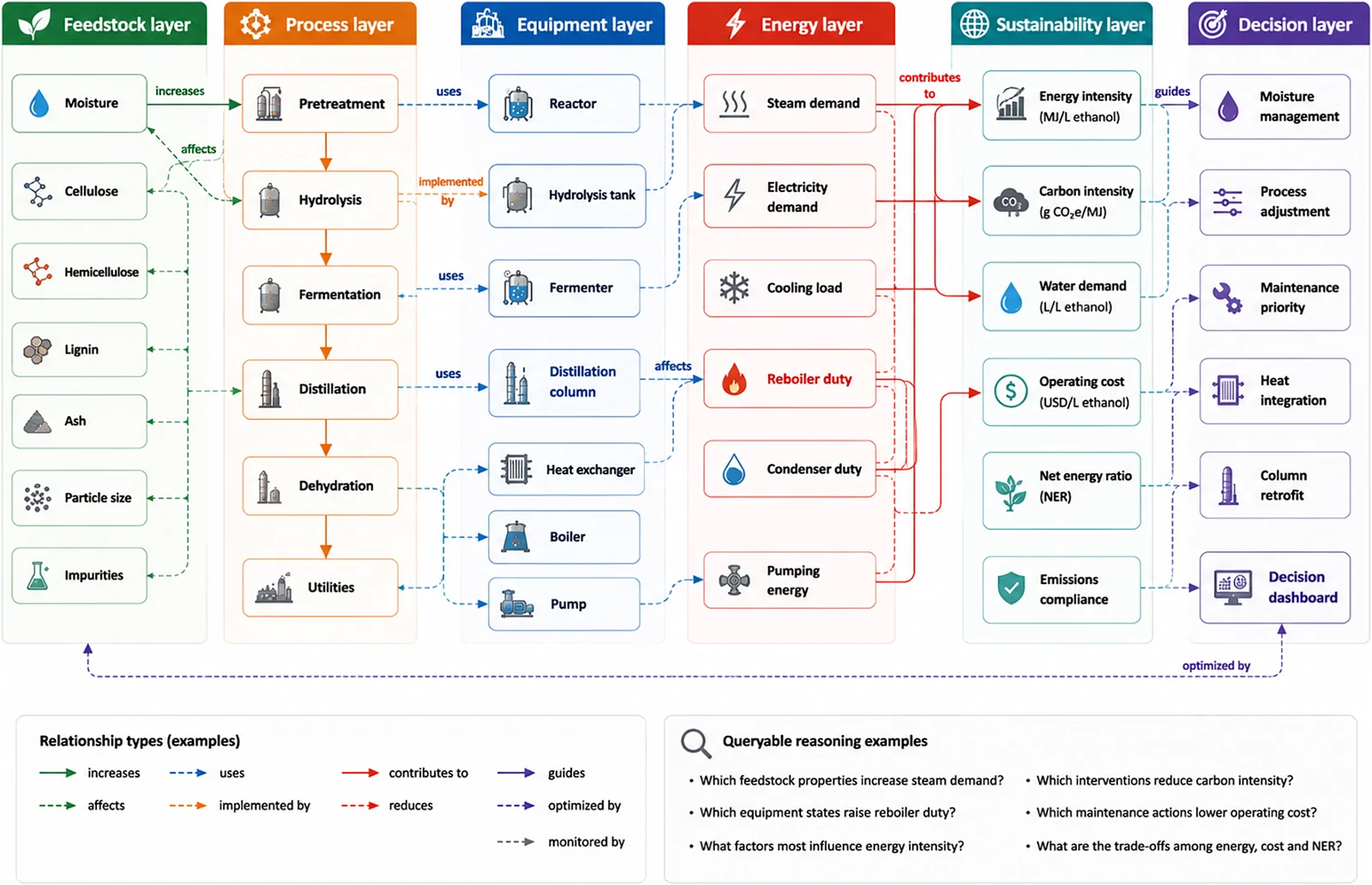
Knowledge-graph ontology for low-carbon bioethanol energy management.

### 3.2 Metric System for Decision Support

A KG–DT framework requires a metric system that defines which variables should be represented, how they are connected, and how they support decisions. In bioethanol studies, metrics are often reported within separate analytical contexts: feedstock studies emphasize moisture, composition, ash, and particle size; pretreatment studies report severity, solid recovery, sugar release, and inhibitor formation; fermentation studies focus on ethanol titer, yield, productivity, and residual sugars; separation studies report reboiler duty, ethanol recovery, product purity, and dehydration demand; TEA and LCA report cost, carbon intensity, fossil energy use, water demand, and coproduct credits. These indicators are individually established in the literature, but KG–DT modeling requires them to be reorganized as connected decision variables rather than isolated end points.

The first metric group describes the process chain. Feedstock metrics define the upstream boundary conditions for the twin because moisture, ash, carbohydrate composition, lignin content, particle size, bulk density, and storage condition affect preprocessing, feeding stability, pretreatment response, and conversion potential.^19,20,93^ Pretreatment and hydrolysis metrics connect these feedstock properties to severity, solid recovery, sugar recovery, inhibitor formation, enzyme loading, digestibility, and viscosity-related processing constraints.^94–96^ Retention-aware pretreatment studies further indicate that compositional enrichment in the recovered solid and absolute component retention on the original-feedstock basis provide different but complementary information.^97,98^ Fermentation and separation metrics then link sugar availability and inhibition to ethanol titer, yield, productivity, residual sugars, ethanol recovery, reboiler duty, dehydration demand, and product purity.^63,84,99^ The important point is that a metric such as ethanol titer should not be stored only as a fermentation output. In a KG–DT system, it is also a driver of separation energy, carbon intensity, utility cost, and intervention priority.

The second metric group connects energy performance with sustainability and economic decision making. Carbon intensity, fossil energy demand, water demand, wastewater load, chemical consumption, and coproduct treatment are needed because a lower process-energy value does not automatically mean lower life-cycle impact or lower cost.^100,101^ Economic metrics such as feedstock cost, enzyme cost, chemical cost, utility cost, maintenance cost, capital intensity, minimum ethanol selling price, and carbon-abatement cost translate technical variation into investment and operating consequences.^102,103^ Heat-integration and exergy metrics add a thermodynamic decision layer by showing whether an energy penalty should be addressed through unit-operation adjustment, heat recovery, combined heat and power, utility-network redesign, or equipment retrofit.^104,105^ Similar assessments in broader energy and resource systems have shown that energy-saving performance, emission reduction, economic outcomes, and resource constraints may need to be evaluated jointly rather than through a single performance indicator.^106–108^ These indicators allow KG–DT reasoning to move from single-objective energy minimization toward multiobjective energy–carbon–cost interpretation.

The third metric group describes the reliability of the digital decision support. Equipment-state metrics are needed to distinguish process-driven energy penalties from degradation-driven penalties. Heat-transfer coefficients, fouling resistance, column pressure drop, tray or packing efficiency, boiler efficiency, pump efficiency, vibration, current, downtime, and maintenance history can indicate whether increased energy use is caused by the process stream itself or by equipment performance loss.^109,110^ Beyond conventional scalar condition-monitoring signals, machine-vision-based approaches in other infrastructure systems demonstrate that equipment-state assessment can also exploit multiscale component detection and few-shot anomaly recognition when abnormal samples are limited or heterogeneous.^111,112^ Multisensor and multimodel condition-monitoring approaches further demonstrate how heterogeneous equipment signals can be combined for degradation assessment and remaining-useful-life prediction.^113^ Data-readiness metrics are equally important because DT reliability depends on the quality, interoperability, uncertainty characterization, and validation of the data used to update the digital representation. Robust perception also requires models to remain reliable when observations are affected by degradation or distribution shifts; degradation-aware learning and knowledge-distillation strategies in other vision-based monitoring tasks provide methodological examples of improving detection robustness under imperfect input conditions.^114^ Representative indicators for the proposed framework therefore include sensor coverage, update frequency, missing-data rate, metadata completeness, measurement uncertainty, semantic consistency, interoperability, and model validation status.^115–117^ This layer gives each model output a confidence context and helps determine whether the system should recommend an intervention, request additional measurements, recalibrate the twin, or fall back to conservative operating rules.

Table 2 reorganizes literature-derived indicators into metric groups that can support KG–DT-based decision making. The table separates process-chain metrics, sustainability and economic metrics, equipment-state metrics, data-readiness metrics, and heat-integration metrics, but these categories are intended to be connected in the knowledge graph. This organization allows a query such as “why did total energy intensity increase this week?” to retrieve linked evidence from feedstock moisture, ethanol titer, column efficiency, reboiler duty, carbon intensity, and maintenance history rather than returning a single isolated indicator.

**Table 2:** Literature-Derived Metric Groups for Adaptive Low-Carbon Bioethanol Energy Management

metric group	representative indicators	typical data source	reported use in literature	role in KG–DT decision support	refs
Feedstock variability metrics	Moisture content; cellulose, hemicellulose, and lignin contents; ash and inorganic elements such as Si, K, and Cl; particle size; bulk density; storage time; degradation state	Online moisture sensors; near-infrared spectroscopy; laboratory compositional analysis; storage records; milling and screening records; LIMS	Feedstock moisture has been used to explain variation in pretreatment heat transfer, steam demand, and ethanol yield. Structural composition is used to estimate theoretical sugar and ethanol potential, whereas ash and inorganic elements are linked to fouling, corrosion, and chemical neutralization demand. Particle size and bulk density affect size-reduction energy, feeding stability, and logistics.	Defines upstream KG nodes and prior causal drivers for feedstock grading, blending, storage prioritization, and recipe switching. In the DT, these metrics set boundary conditions for energy and yield prediction, helping distinguish feedstock-driven energy penalties from equipment-driven inefficiency.	^19,20,59,76,118^
Pretreatment and hydrolysis performance metrics	Severity factor, such as log R0; solid recovery; water-insoluble solids; glucan and xylan conversion; inhibitor concentrations, such as furfural, HMF, acetic acid, and phenolics; enzyme loading; liquid-to-solid ratio; residence time	Pretreatment reactor historian; online temperature, pressure, and flow data; HPLC, IC, or GC measurements; enzyme dosing records; solids analysis; enzymatic digestibility tests	Severity factor is widely used as a unified index for comparing pretreatment conditions. Sugar recovery and enzymatic digestibility indicate whether higher pretreatment energy and chemical input are justified. Inhibitor concentrations are used to assess fermentation risk, detoxification requirements, and sugar-loss trade-offs.	Connects pretreatment severity, inhibitor formation, enzyme digestibility, fermentation risk, and downstream separation burden in the KG. In the DT, these metrics support adaptive adjustment of steam input, chemical loading, residence time, enzyme dosage, and detoxification strategies.	^6,22,119–121^
Fermentation and product-concentration metrics	Ethanol titer; ethanol yield; volumetric productivity; residual glucose and xylose; xylose utilization; cell viability; inhibitor tolerance; fermentation time; CO_2_ evolution rate	Fermenter pH, temperature, ORP, and off-gas data; soft sensors; HPLC or GC; biomass/cell-count measurements; batch records; PAT data	Ethanol titer is repeatedly used as a bridge metric linking fermentation performance to downstream distillation energy. Yield and productivity determine unit productivity and downstream load per unit ethanol. Residual sugars and xylose utilization identify incomplete conversion and sugar-loss mechanisms.	Enables the DT to predict harvest timing, distillation load, and energy per liter of ethanol. In the KG, fermentation metrics link inhibitors, strain performance, nutrient conditions, and product concentration, supporting high-solids and high-titer operating strategies.	^63,84,122–124^
Separation and purification energy metrics	Reboiler duty; condenser duty; ethanol recovery; reflux ratio; column pressure; number of theoretical stages; dehydration energy; molecular-sieve regeneration load; product purity; ethanol loss in bottoms	Steam and cooling-water meters; column temperature and pressure profiles; Aspen Plus models; molecular-sieve cycle logs; online density or concentration meters; DCS historian	Separation studies commonly use reboiler duty, recovery, and product purity to compare purification designs. Lower fermentation ethanol concentration generally increases specific distillation and dehydration energy. Recent work has explored sidestreams, heat pumps, pass-through distillation, adsorption, and intensified dehydration to reduce energy demand.	Forms the main energy-penalty layer in the KG–DT framework. Once ethanol titer declines or column efficiency deteriorates, the DT can propagate this effect to reboiler duty, carbon intensity, and operating cost. These metrics support reflux optimization, heat-pump operation, regeneration scheduling, and purity–recovery–energy trade-off analysis.	^6,63,88,99,125,126^
Plant-level energy metrics	Total heat demand; steam demand; electricity demand; cooling demand; CHP contribution; surplus electricity export; specific energy consumption; total energy demand in MJ/L ethanol; net energy ratio; energy return on investment	Utility meters; boiler and turbine data; steam balance; heat-exchanger network models; Aspen or TEA models; SCADA historian	NREL design cases and process-integration studies use these indicators to compare process routes, scales, and integration levels. Steam demand is often the dominant utility load in biochemical ethanol systems, while CHP and lignin/residue combustion strongly affect external energy demand.	Provides the main interface between unit-level twins and the plant-level DT. These metrics support boiler–turbine–distillation–evaporation coordination, plant dashboarding, and integrated energy–carbon–cost optimization.	^3,5,6,13,56,127^
Equipment-state and maintenance metrics	Overall heat-transfer coefficient; fouling resistance; column pressure drop; tray or packing efficiency; flooding margin; boiler efficiency; excess air; flue-gas loss; pump or compressor efficiency; vibration; current; MTBF; downtime; maintenance backlog; sensor drift; alarm frequency	Vibration and motor-current sensors; DCS historian; CMMS/EAM systems; condition-monitoring systems; inspection records; online heat-balance and pressure-drop data	Process-industry DT and predictive-maintenance studies use these metrics as proxies for equipment degradation, early fault formation, and energy-efficiency loss. Heat-exchanger fouling raises steam demand, column deterioration increases separation energy, and boiler efficiency directly affects plant-level carbon intensity.	Allows KG–DT outputs to trace energy anomalies to equipment states rather than producing black-box alarms. These metrics support maintenance prioritization, cleaning scheduling, boiler inspection, spare-parts planning, and load-reduction decisions.	^24,25,27,64,128,129^
Carbon and resource sustainability metrics	Life-cycle carbon intensity; fossil energy demand; petroleum displacement; freshwater use; wastewater COD/BOD; nitrogen and phosphorus load; acid, alkali, enzyme, and nutrient inputs; residue utilization; coproduct credits	GREET or LCI databases; plant mass and energy balances; water and wastewater records; emission factors; process simulation; LCA software	LCA studies use GHG emissions, fossil energy, and water use to compare routes and feedstocks. Chemical inputs, steam demand, coproduct allocation, and system-boundary choices can substantially change conclusions. Regional studies also show that background energy mix and chemical supply chains affect carbon performance.	Extends KG–DT optimization from energy saving to low-carbon and resource-aware operation. These indicators support carbon-aware control, low-carbon feedstock selection, compliance reporting, water-use management, and explainable emission-reduction evidence chains.	^3,4,13,14,34^
Economic and decision metrics	Minimum ethanol selling price; minimum selling price; CAPEX; total capital investment; OPEX by feedstock, enzyme, chemical, utility, and maintenance cost; IRR; NPV; payback period; carbon-abatement cost; shadow carbon price; sensitivity ranking; uncertainty ranking	TEA spreadsheets; Aspen-linked cost models; market-price databases; financial assumptions; carbon-price scenarios; sensitivity and uncertainty-analysis outputs	TEA literature uses MESP, CAPEX, and OPEX to compare process routes and scales. Feedstock cost, plant scale, ethanol yield, enzyme cost, and utility prices are commonly identified as high-sensitivity variables. Recent studies emphasize linking economic metrics with sustainability and coproduct value.	Provides the final action-ranking layer in KG–DT decision support. It translates changes in energy demand, carbon intensity, and equipment condition into financial consequences, enabling intervention prioritization, retrofit evaluation, recipe switching, and profit optimization under carbon constraints.	^5,6,12,33,58,130^
Data-quality and readiness metrics	Sensor coverage; sampling frequency; update frequency; timestamp synchronization; missing-data rate; data completeness; measurement uncertainty; repeatability; metadata completeness; ontology completeness; interoperability; validation status; concept drift	Historian systems; sensor QA/QC; data dictionaries; ETL logs; ontology and KG models; validation records; retraining logs; model-monitoring systems	DT and KG literature treats data availability, validation, interoperability, and model trust as prerequisites for deployment. Missing data, drift, and clock mismatch weaken diagnosis, prediction, and optimization reliability. KG studies emphasize semantic consistency and relationship completeness across heterogeneous data sources.	Determines the trust layer of the KG–DT framework. These metrics allow each decision output to carry a confidence score, trigger fallback logic, guide soft-sensor selection, define recalibration frequency, and determine whether the system should be used for screening, calibrated prediction or plant-linked decision support.	^27,30,31,53,64,65^
Heat-integration and exergy metrics	Exergy efficiency; heat-recovery ratio; Δ*T* min; pinch margin; waste-heat utilization; CHP contribution; external utility replacement; heat-pump duty; retrofit potential	Aspen or pinch models; steam and heat-exchanger network data; boiler/turbine records; plant energy maps; TEA and LCA coupled models	Heat-integration and exergy studies use these metrics to identify thermodynamic inefficiencies and utility waste. They are especially useful for explaining why plants with similar ethanol yield may show different steam demands. These metrics support evaluation of heat pumps, sidestream distillation, process restructuring, and retrofit options.	Functions as a cross-unit coupling layer linking pretreatment, fermentation, distillation, evaporation, and CHP. In KG–DT optimization, these metrics help minimize external steam demand, carbon intensity, and total cost while ranking retrofit opportunities.	^16,56,99,125,127^

### 3.3 Coupling KG with DT

Coupling a knowledge graph with a digital twin requires a clear division of the function. The KG provides semantic structure, causal traceability, queryable relationships, and rule representation, whereas DT provides dynamic simulation, state updating, scenario testing, anomaly detection, and optimization. Industrial DT studies commonly define the twin as a synchronized digital representation that uses data and models to monitor, predict, and optimize the behavior of a physical asset or process.^131,132^ Recent reviews further emphasize that scalable DTs require not only sensors and models but also model updating, information exchange, interoperability, and uncertainty handling.^133,134^ In the bioethanol context, this means that the KG should define what each variable means and how variables are related, while the DT should estimate how the system state changes when feedstock, process, equipment, or utility conditions vary. Physics-informed learning in other dynamic engineering systems provides a related methodological precedent, showing that machine-vision features can be coupled with physical constraints and multiscale dynamic models to improve state estimation while retaining greater physical interpretability than purely data-driven prediction.^135^

The information flow should therefore be bidirectional rather than a one-way transfer from the plant data to a simulator. Feedstock, process, equipment, and utility data first enter the KG as semantically defined entities with units, time stamps, provenance, uncertainty, and links to relevant process nodes. The DT then uses validated inputs from the KG to simulate energy demand, product concentration, separation load, equipment-state effects, and carbon or cost consequences. The simulated outputs return to the KG as the updated states and evidence for diagnosis. This structure allows the decision layer to distinguish causes that may look similar in the aggregate energy data. For example, if feedstock moisture increases, the KG can identify related pretreatment, hydrolysis, separation, and energy nodes, while the DT can estimate additional heating demand and the downstream separation burden. If column efficiency declines, the DT can estimate the resulting increase in reboiler duty, while the KG links this change to its implications for carbon intensity, maintenance priority, and possible operating adjustments. This loop turns energy prediction into traceable decision support, rather than isolated numerical forecasting. Comparable decision-support applications in other resource-management systems have likewise used model-based analysis to translate system states and operational constraints into management actions.^136,137^ More recent data-science workflows further demonstrate that feature screening, interpretable machine learning, and multiobjective optimization can be combined with mechanistic analysis to convert prediction into explainable design decisions.^138,139^

As shown in Figure 3, KG–DT implementation can progress from an offline literature-curated framework to pilot-scale calibration, plant-linked adaptive monitoring, and low-carbon decision optimization. In the first stage, literature values, expert rules, process assumptions, and benchmark models can be used to build the ontology, define causal links, and test offline scenarios. This stage is useful for structuring knowledge and identifying the variables most likely to influence energy intensity, even before extensive plant data are available. In the second stage, pilot-scale or demonstration-scale data can calibrate key parameters, quantify uncertainty, and test whether the assumed causal links remain valid under controlled variation. In the third stage, plant-linked twins can use sensor streams, utility meters, laboratory measurements, and maintenance records to update system states and diagnose deviations. In the final stage, the framework can support optimization across energy, carbon, cost, equipment health, and heat-integration objectives. This staged pathway is consistent with the broader DT literature, which treats full real-time autonomy as a mature deployment state rather than an initial modeling requirement.^140,141^

**Figure 3:**
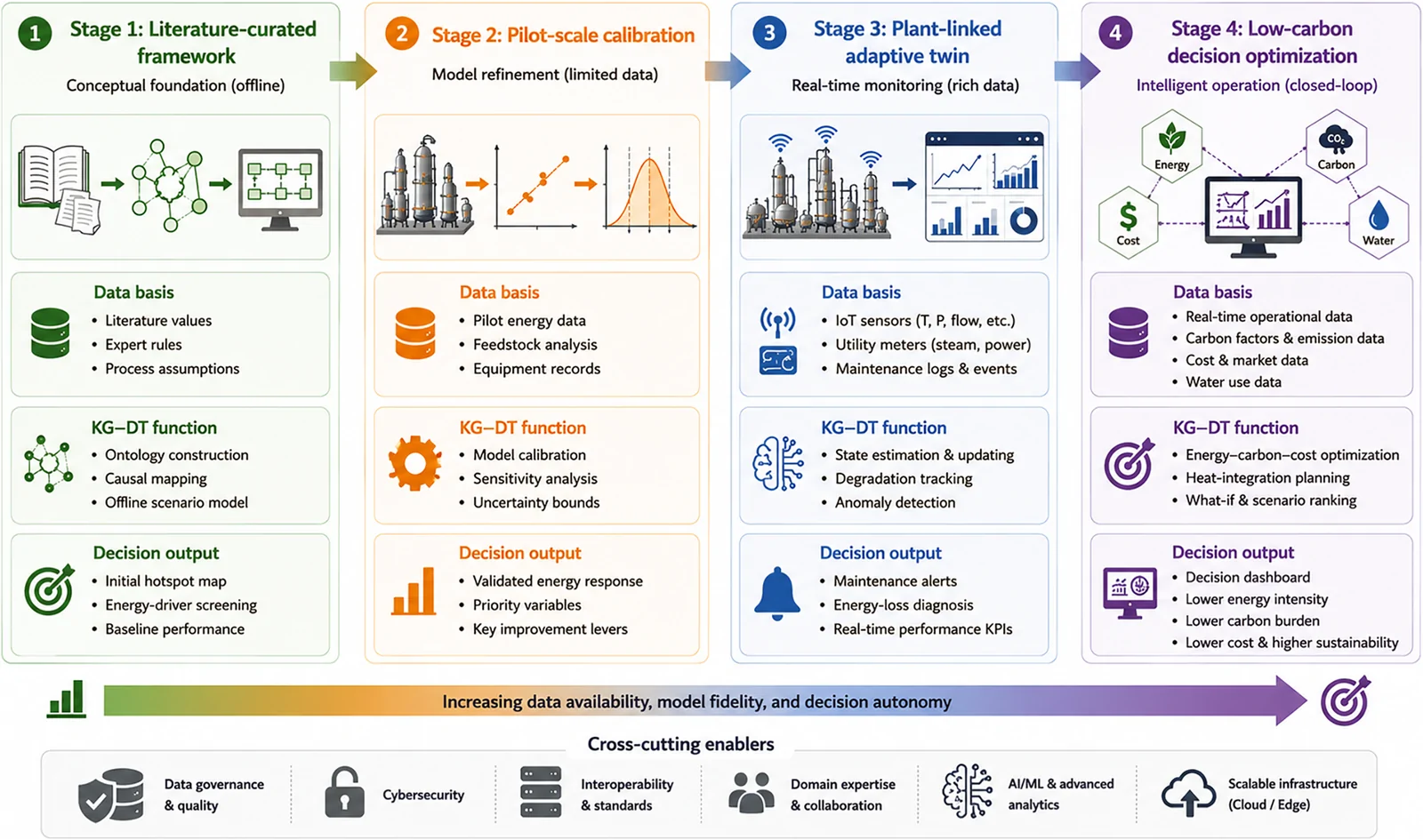
Implementation pathway from offline KG–DT framework to plant-linked adaptive energy management.

Deployment of KG–DT systems also depends on data and governance conditions that are often underestimated in conceptual frameworks. Sensor coverage, metadata consistency, data synchronization, validation status, cybersecurity, and access control determine whether model outputs can be trusted in operational decisions. Recent DT surveys identify security, privacy, interoperability, real-time communication, model fidelity, and life-cycle maintenance as persistent implementation challenges.^142,143^ The reliability of the sensing and communication layer is another prerequisite for trustworthy plant-linked digital twins. Reliability studies of wireless sensor networks show that local sensor failures, relay dependence, isolation effects, and propagated failures can interact over time, making system-level data availability dependent not only on individual sensor performance but also on network structure and functional dependencies. Knowledge-graph-based DT studies further show that semantic links can help integrate heterogeneous data sources and simulation models, but they also require maintenance of vocabularies, mappings, and model relationships as the plant changes over time.^29^ KG–DT should therefore be viewed as a controlled learning infrastructure rather than a plug-in digital product. Its purpose is not to replace process knowledge with automation but to connect process knowledge, operational data, simulation, and decision logic in a form that can be inspected, updated, and validated. Before such a system can support plant-level decision making, however, the additional requirements of KG–DT deployment need to be considered against the operational and economic value that they are expected to generate.

### 3.4 Economic Feasibility of KG–DT Deployment

Economic feasibility of KG–DT deployment should be evaluated separately from the conventional techno-economic feasibility of lignocellulosic ethanol production. Conventional TEA addresses the economics of the production pathway itself by relating capital investment and operating expenditure to feedstock cost, enzyme and chemical consumption, utility demand, conversion yield, and ethanol selling price.^12,33,58,130^ A KG–DT system introduces an additional cost layer that arises from the digital infrastructure required to observe, represent, model, and continuously update the physical process, including instrumentation, data integration, model development, semantic maintenance, computing, and life-cycle support.^144,145^ The economic question is therefore narrower and more operational: whether a defined KG–DT application can recover these additional life-cycle costs by reducing a measurable source of process loss, such as excessive utility consumption, equipment-related efficiency deterioration, unplanned downtime, repeated operating trials, or production losses.^146–148^ This balance depends on the scope and fidelity of the twin as well as the economic consequence of the problem being addressed; increasing sensor coverage, model resolution, or system connectivity is not economically justified when the additional information does not produce a corresponding improvement in operational decisions. The appropriate deployment boundary should therefore be determined by the value of the targeted use case rather than by the technical possibility of constructing a plant-wide twin. Bioethanol-specific digital-twin studies currently provide development pathways and bench-scale demonstrations, including second-generation ethanol fermentation, but do not yet provide the plant-scale investment evidence needed to define a general return on KG–DT deployment.^64^ Economic feasibility in this context should consequently be assessed as the incremental value of a defined digital intervention relative to its implementation and life-cycle cost, while conventional TEA remains responsible for evaluating the underlying ethanol-production pathway.

The cost of KG–DT deployment is distributed across the physical–digital interface, the modeling infrastructure, and the subsequent operation of the digital system. At the plant interface, additional expenditure may arise from process and utility metering, equipment-condition monitoring, data-acquisition hardware, sensor installation or retrofit, and the calibration and replacement needed to maintain the measurement quality.^144,145,149^ These requirements are strongly affected by the instrumentation already available at the plant because connecting existing historians, DCS, SCADA, laboratory systems, and maintenance records can require substantial data mapping, synchronization, and interface development before the information is usable by a twin. The semantic and modeling layers create a different cost burden. Ontology development requires process variables, equipment, models, units, relationships, and provenance to be represented consistently, while the DT itself requires model construction, parameter estimation, calibration, validation, and computing resources. These activities also continue after initial deployment: process changes, new feedstocks, equipment modifications, sensor replacement, and model drift can require updates to mappings, parameters, rules, and validation records.^28,29^ Life-cycle expenditure therefore includes data storage and processing, software and computing resources, sensor maintenance, model recalibration, ontology maintenance, cybersecurity, governance, and personnel capable of maintaining both process and digital knowledge.^142,150,151^ For this reason, the economically relevant cost is the total cost of maintaining a usable KG–DT over its intended service period, rather than the one-time cost of creating its initial digital representation.

The economic benefit of KG–DT deployment is most readily demonstrated when digital decisions can be linked to losses already visible in plant energy, production, and maintenance accounts. Direct benefits can arise from lower steam, electricity, and fuel consumption, reduced degradation-related utility penalties, fewer unplanned interruptions, and improved production or product recovery.^25,128,129^ These effects can be translated into monetary terms using utility tariffs, maintenance expenditure, downtime losses, production margins, and, where applicable, the cost assigned to carbon emissions. Industrial applications have shown that such benefits can be measurable, although their magnitude is strongly process- and use-case-dependent: energy-oriented DT implementations have reported energy reductions of up to 40% in a heating operation, approximately 16% lower natural-gas consumption in an industrial spray-drying application, and about 12% reductions in energy use and associated GHG emissions in an optimized dyeing process.^146–148^ These values should not be transferred directly to lignocellulosic ethanol plants, but they demonstrate that energy and carbon benefits can be quantified against an operating baseline rather than treated only as qualitative expectations. In bioethanol production, economically relevant applications are most readily identified where energy or availability losses are both substantial and measurable. Distillation and dehydration are sensitive to fermentation titer and separation performance,^63,84,99^ while heat-exchanger fouling and declining equipment efficiency can increase steam demand and maintenance requirements.^89,90^ Linking these losses to equipment condition, process state, and intervention history would allow KG–DT benefits to be expressed as avoided utility expenditure, avoided downtime, recovered production, or deferred maintenance cost. Other benefits, such as faster diagnosis, improved reuse of process knowledge, and reduced trial-and-error, may also contribute to economic performance but require plant-specific valuation because they are less directly reflected in conventional operating accounts.^152,153^ Cross-domain applications of intelligent design and automated manufacturing further show that digital parametrization can connect design decisions with cost control, production efficiency, and sustainability considerations before physical implementation.^154^ Table 3 brings these cost and benefit pathways together with the corresponding evaluation indicators and deployment stages, providing a basis for assessing where KG–DT investment can be economically justified rather than assuming that full-system deployment is inherently advantageous.

**Table 3:** Implementation Costs, Potential Benefits, and Economic Evaluation Metrics for Staged KG–DT Deployment in Bioethanol Energy Management^a^

KG–DT deployment element	principal implementation and recurring costs	main operational and economic benefit pathways	decision-relevant economic and performance indicators	relevant deployment stage	refs
Sensing and data acquisition	Installation or retrofit of process, utility, and equipment-condition sensors; flow, steam, electricity, temperature, pressure, moisture, and composition measurements; data-acquisition hardware; sensor calibration, replacement, and routine maintenance	Improved visibility of feedstock, process, equipment, and utility states; earlier detection of deviations and energy losses; improved boundary conditions for DT state estimation; reduced uncertainty in energy attribution	Sensor and metering CAPEX; calibration and maintenance OPEX; measurement availability and completeness; detected utility loss; avoided energy cost; value of additional information	Pilot calibration → plant-linked monitoring	^144,145,149^
Data infrastructure and interoperability	Historian and database expansion; data pipelines; interfaces with DCS, SCADA, LIMS, CMMS/EAM, and laboratory systems; data synchronization and standardization; communication infrastructure; integration of legacy and newly instrumented assets	Reduced fragmentation among process, utility, laboratory, and maintenance data; reduced manual reconciliation; faster cross-layer diagnosis; reusable and scalable DT services across units and assets	Integration CAPEX; annual data-management OPEX; data latency; synchronization quality; interoperability coverage; proportion of connected assets; labor or engineering time required for data reconciliation	Offline/pilot framework → plant-linked monitoring	^28–31,155^
Knowledge-graph and ontology layer	Ontology development; definition of entities, attributes, and relationships; semantic mapping of heterogeneous data; knowledge curation and validation; rule construction; maintenance of vocabularies, mappings, and model relationships	Improved semantic interoperability and knowledge reuse; explicit links among feedstock, process, equipment, energy, sustainability, and decision entities; improved causal traceability and explainability; more efficient model discovery and configuration	Ontology-development and maintenance effort; mapping completeness; percentage of connected variables or assets; query and diagnosis time; reusable model or rule coverage; frequency of ontology updating	Offline framework → continuous life-cycle use	^28,29,156^
DT model development, calibration, and life-cycle maintenance	Development of mechanistic, data-driven, or hybrid models; parameter estimation; model validation; software and engineering labor; computing resources; model recalibration, version control, and life-cycle updating	Dynamic state estimation; scenario testing; prediction of process and energy consequences before physical intervention; reduced trial-and-error; improved operating-window selection; support for plant-wide process optimization	Model-development and recalibration cost; computation and software OPEX; prediction error and uncertainty; update frequency; avoided experimental or operating trial cost; value of improved decision accuracy	Pilot calibration → plant-linked monitoring and optimization	^64,152,153^
Predictive maintenance and equipment-state analytics	Condition-monitoring instrumentation; degradation, fault-diagnosis, and remaining-useful-life models; maintenance-system integration; additional validation and model updating	Earlier fault identification; improved maintenance and cleaning schedules; reduced unplanned downtime; avoidance of persistent energy penalties caused by fouling, reduced heat transfer, pumping inefficiency, or separation-performance deterioration	Avoided downtime value; maintenance expenditure; MTBF; equipment availability; degradation-related utility cost; maintenance interval; efficiency recovered after intervention	Plant-linked monitoring → adaptive optimization	^25,128,129,157^
Energy, utility, and low-carbon optimization	Energy-model integration; optimization and control algorithms; bidirectional plant–model communication; operator interfaces; testing and validation of operating recommendations; integration with heat- and utility-management systems	Reduced steam, electricity, and fuel use; improved heat and utility integration; higher process efficiency and production stability; improved asset utilization; lower energy-related GHG emissions and utility expenditure	Specific energy consumption; annual steam and electricity savings; fuel-cost reduction; production or recovery improvement; carbon-intensity reduction; avoided carbon cost where applicable; operating-cost reduction	Plant-linked monitoring → low-carbon decision optimization	^146–148^
Cybersecurity, governance, and organizational capability	Cybersecurity architecture and access control; secure data exchange; backup and version management; data and model governance; documentation; specialist personnel; operator and engineering training; continuing maintenance of digital assets	Increased reliability and traceability of digital decisions; reduced cyber and data-integrity risk; sustained model validity; greater user trust and organizational adoption; improved capability to maintain the KG–DT system over its life cycle	Annual cybersecurity and governance OPEX; system availability; security incident frequency; data/model auditability; personnel and training cost; model and ontology maintenance effort; user adoption or utilization rate	All stages, with increasing requirements as connectivity and automation increase	^142,150,151^
Investment appraisal and staged scale-up	Incremental investment associated with greater sensing coverage, data connectivity, semantic integration, model fidelity, computing capacity, and plant-level synchronization; additional costs of integrating legacy systems and sustaining the DT throughout its life cycle	Identification of economically justified DT scope and fidelity; avoidance of overengineering; prioritization of high-value assets and energy hotspots; staged realization of energy, maintenance, production, and carbon benefits; improved justification for brownfield retrofit or greenfield integration	Annual net benefit; ROI; simple payback period; NPV; IRR; benefit–cost ratio; cost per unit energy saved; cost per unit CO_2_-equivalent avoided; sensitivity of economic returns to energy price, plant scale, downtime, digital maturity, and model fidelity	Decision gate between offline, pilot, plant-linked, and optimization stages	^158–162^

a CAPEX, capital expenditure; CMMS, computerized maintenance management system; DCS, distributed control system; DT, digital twin; EAM, enterprise asset management; IRR, internal rate of return; KG, knowledge graph; LIMS, laboratory information management system; MTBF, mean time between failures; NPV, net present value; OPEX, operating expenditure; ROI, return on investment; SCADA, supervisory control and data acquisition.

Economic appraisal should be based on the incremental cash flow attributable to the KG–DT application rather than on the overall profitability of the ethanol plant. The appropriate baseline is therefore the same process operated without the proposed digital intervention, so that savings in utilities, maintenance, downtime, or production losses are counted only when they can reasonably be attributed to improved monitoring, diagnosis, prediction, or optimization.^158–162^ Initial implementation expenditure and recurring digital OPEX should then be evaluated against these incremental benefits over the intended service life of the system. Net present value and internal rate of return are particularly suitable for this comparison because they account for the timing of costs and benefits and allow alternative DT configurations to be compared on a common investment basis, while payback period and ROI can provide simpler screening indicators for early-stage deployment decisions.^158,159^ Because both costs and realized benefits are uncertain before plant implementation, a single deterministic return is unlikely to be sufficient. Probabilistic approaches such as Monte Carlo analysis provide one route for examining how uncertainty in key inputs propagates into decision outcomes.^163^ Investment performance should therefore be tested against variables that directly affect the value of the targeted use case, including steam and electricity prices, baseline energy intensity, frequency and cost of unplanned downtime, maintenance requirements, plant throughput, existing sensor and data infrastructure, model-update requirements, system lifetime, and discount rate.^158,160–162^ Carbon-related savings can be included where an explicit carbon price, internal carbon value, or regulatory cost is available, but should be kept separate from physical emission reductions when no monetary value can be assigned. Broader energy-economic assessments have also monetized external health and environmental costs and the avoided external costs associated with cleaner energy systems, providing complementary approaches for valuing benefits that are not directly reflected in plant utility expenditure.^164,165^ The economic context of low-carbon technology deployment may also be affected by policy incentives and regulatory conditions because government subsidies, market-based environmental regulation, and environmental taxation can alter technology-investment and innovation incentives.^166–168^ A more detailed or more highly connected twin is economically preferable only when the additional value created by greater prediction accuracy, broader process coverage, or faster decision support exceeds the corresponding increase in development and life-cycle cost.^158,159^ The investment decision is therefore not whether a KG–DT system is beneficial in general but which level of digital capability provides the highest defensible value for the operational problem being addressed.

Economic feasibility therefore favors staged rather than plant-wide KG–DT deployment. Initial implementation should focus on process units or operating problems for which energy, maintenance, or production losses are both measurable and economically significant, while further expansion should depend on demonstrated data quality, model reliability, and realized operational value. At a broader scale, digitalization has been examined in relation to both energy efficiency and carbon performance, but such environmental gains should be demonstrated for the specific implementation context rather than assumed from digital adoption alone.^169,170^ In this way, the implementation pathway shown in Figure 3 can also serve as an economic decision sequence, allowing digital capability to expand only when additional investment is supported by measurable benefits.

## 4 Illustrative Decision Map: From Prediction to Intervention

Energy prediction has limited operational value if it only reports that total energy demand has increased. For adaptive bioethanol management, the more important question is why the penalty occurred and which response should be prioritized. A similar increase in energy intensity may result from wet feedstock, low sugar conversion, poor fermentation performance, separation inefficiency, heat-exchanger fouling, or weak utility integration. These causes are not equivalent because they require different interventions. A KG–DT framework is therefore useful only when it can connect predicted energy values with causal evidence and action logic. The illustrative decision map in this section is not intended to validate a specific industrial plant. It is used as a conceptual demonstration of how coupled operating disturbances can be interpreted within the proposed framework.

Feedstock moisture and distillation efficiency were selected as the two scenario variables because they represent different layers of the bioethanol system. Moisture is an upstream material property that can alter the heating demand, slurry handling, solids loading, pretreatment response, and downstream product concentration. Distillation efficiency is a downstream equipment-state variable that affects reboiler duty, ethanol recovery, separation cost, and carbon burden. In the illustrative scenario, the baseline case is defined as 20% feedstock moisture and 75% distillation efficiency, corresponding to a total energy demand of 12.0 MJ/L of ethanol. A high-moisture case is defined as 40% moisture with an unchanged distillation efficiency, increasing the total energy demand to 14.0 MJ/L. A degraded-efficiency case is defined as 20% moisture and 60% distillation efficiency, increasing the total energy demand to 13.8 MJ/L. These values are used to demonstrate decision logic rather than to represent plant-validated measurements.

As shown in Figure 4, the illustrative decision map combines an energy-response surface with intervention zones so that moisture-driven and equipment-driven penalties can be distinguished. The stable operation zone corresponds to low moisture and acceptable distillation efficiency, where routine monitoring, heat-recovery fine-tuning, and normal process control are sufficient. The moisture-driven penalty zone indicates that increased energy demand is mainly associated with feedstock conditions; suitable responses include feedstock drying, blending, storage control, and solids-loading adjustment. The equipment-driven penalty zone indicates that the main evidence points to separation inefficiency; suitable responses include column inspection, fouling control, reboiler and condenser diagnosis, maintenance scheduling, and efficiency recovery. The combined high-risk zone occurs when high moisture and low distillation efficiency interact, requiring coordinated intervention rather than a single unit adjustment.

**Figure 4:**
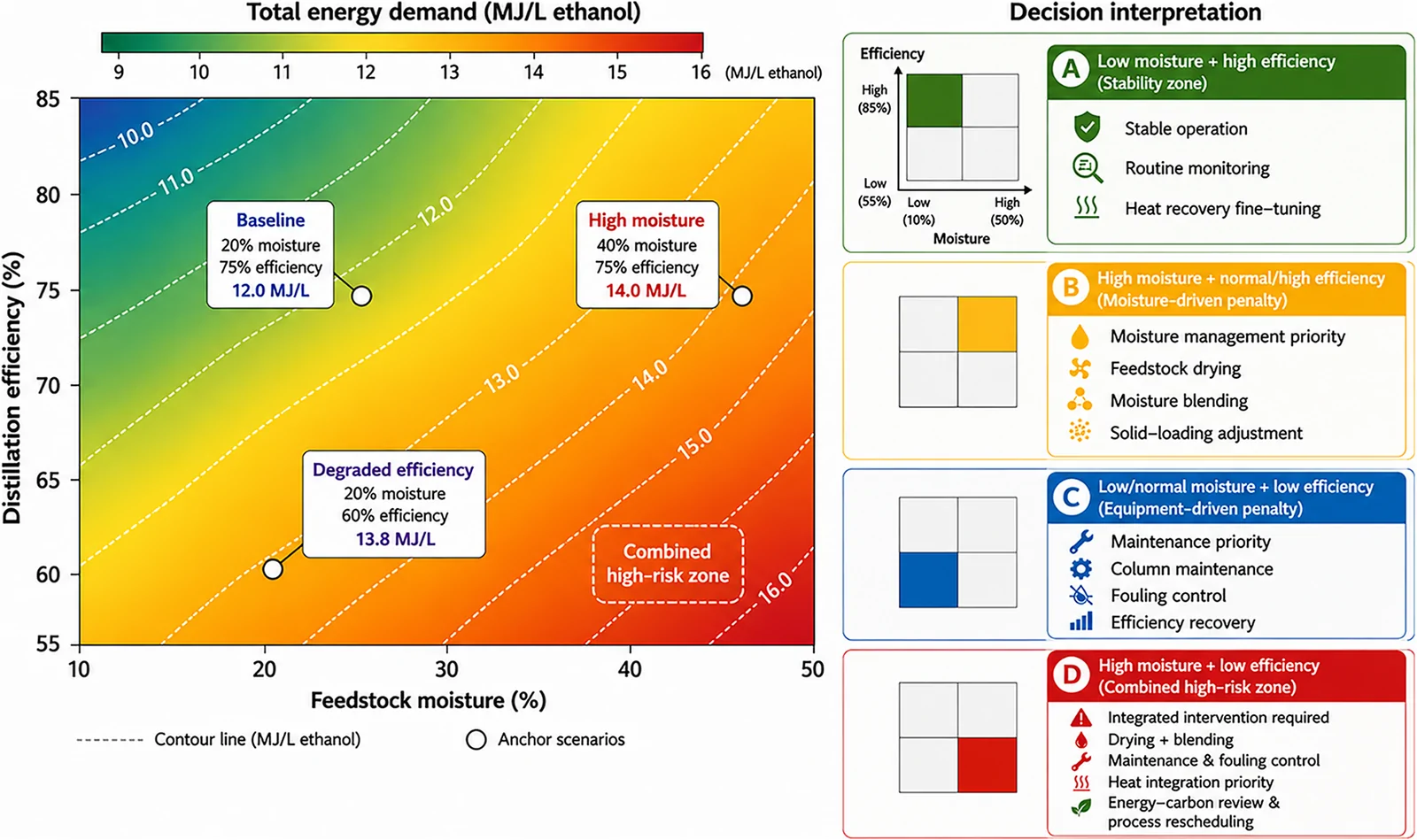
Illustrative decision map linking feedstock moisture and distillation efficiency to energy-management priorities.

The value of this map is that it separates energy magnitude from intervention meaning. A total energy demand near 14 MJ/L may appear similar in aggregate accounting, but the decision response differs depending on whether the evidence points to wet biomass, column inefficiency, or both. In a KG–DT system, this distinction is made through linked entities and model outputs: feedstock moisture is connected to pretreatment heat demand and solids handling; ethanol titer is connected to distillation load; column efficiency is connected to reboiler duty and maintenance state; and all of these variables are connected to carbon intensity, utility cost, and decision priority. The same energy value can therefore be interpreted through different causal routes. In plant application, these alternative causal routes could also be differentiated economically by linking the predicted energy penalty to utility expenditure, intervention cost, downtime risk, and expected recovery of process performance.

This illustrative case also shows why KG–DT reasoning should not be reduced to numerical optimization. A purely predictive model may estimate that energy demand will increase under high-moisture or low-efficiency conditions, but it may not explain whether the appropriate action is feedstock blending, pretreatment adjustment, fermentation diagnosis, column maintenance, or heat-integration redesign. The KG provides the semantic links needed to interpret the source of the penalty, while the DT estimates the dynamic effect of the disturbance. Together, they convert energy prediction into differentiated intervention priorities. The decision map therefore reinforces the central argument that adaptive low-carbon bioethanol management requires models capable of connecting energy values with causal evidence and operational response.

## 5 Conclusions and Outlook

The central value of KG–DT integration is not to replace established energy-balance, TEA, LCA, process-simulation, or heat-integration methods. Its value is to connect their outputs in a form that supports adaptive interpretation. Conventional assessment can show how much energy was consumed, which unit operation is energy-intensive, or which scenario has a lower carbon burden. A KG–DT system should go further by asking why energy intensity changed, whether the penalty is feedstock-driven or equipment-driven, and which operational response is most defensible. This shift is important because digital-twin research increasingly emphasizes synchronization, state updating, interoperability, and decision support rather than static digital representation alone.

The first opportunity is explainable cross-layer diagnosis. In bioethanol plants, energy penalties often emerge through chains of causes rather than single variables. Wet feedstock may increase heating demand and change solids handling; poor hydrolysis may lower fermentable sugar availability; low ethanol titer may increase distillation duty; heat-exchanger fouling may raise steam consumption; and column inefficiency may increase reboiler load. KG–DT integration can connect these signals through semantic relationships and dynamic models so that diagnosis precedes optimization. This is essential because an optimizer that minimizes an energy objective without causal context may recommend an action that is numerically attractive but operationally inappropriate. Knowledge-graph studies in digital-twin systems highlight this role of semantic links in connecting heterogeneous data sources, simulation models, and decision logic, especially when scalable model configuration and interpretation are required.

The second opportunity is multiobjective low-carbon decision making. Lower process energy does not automatically imply lower life-cycle carbon, lower cost, lower water demand, or higher reliability. A change in pretreatment may reduce downstream enzyme demand but increase chemical use or wastewater burden. A drying strategy may stabilize feedstock and reduce separation energy but add thermal demand if low-grade heat is unavailable. Maintenance may increase short-term downtime but reduce persistent utility losses. A KG–DT framework can make these trade-offs explicit by linking energy intensity, carbon intensity, operating cost, water demand, chemical use, equipment health, and heat-integration potential. In this sense, the framework should not be designed as a single-objective energy minimizer, but as a decision-support structure that allows energy, carbon, cost, and reliability to be interpreted together. Economic feasibility places an additional constraint on this integration. KG–DT deployment should be treated as an incremental investment, with the life-cycle costs of sensing, data integration, model maintenance, and governance weighed against measurable reductions in energy use, maintenance burden, downtime, and production losses. Broader plant integration should therefore proceed only when targeted applications demonstrate sustained operational and economic value.

The main barriers are practical, rather than conceptual. Plant data are often sparse, noisy, proprietary, and unevenly sampled. Laboratory feedstock measurements may not share time stamps with process historian data, and maintenance records may be qualitative or incomplete. Sensor coverage may be sufficient for mass and energy accounting but is insufficient for causal diagnosis. Ontologies also require governance: variable names, units, metadata, relationships, and data provenance must be maintained as equipment, control strategies, and feedstock sources change. Digital-twin surveys consistently identify interoperability, model fidelity, life-cycle maintenance, data exchange, cybersecurity, and privacy as major implementation challenges for industrial systems. These issues are especially important for bioethanol because feedstock supply, process biology, utility systems, and equipment conditions change at different time scales.

Future work should therefore prioritize usable infrastructure rather than overly autonomous claims. Modular ontologies are needed for feedstock, pretreatment, fermentation, separation, utilities, equipment state, carbon accounting, and operational decisions. Open benchmark data sets and reference scenarios would help compare KG–DT methods under common assumptions. Hybrid mechanistic-data-driven twins should be developed because first-principles models are useful for mass and energy consistency, whereas data-driven components are needed for degradation, drift, and soft sensing. Uncertainty-aware dashboards should report not only predicted energy intensity but also confidence, evidence source, causal route, and recommended intervention. Pilot- and plant-scale validation will be essential before KG–DT tools can be used for operational decisions. The near-term goal is not a fully autonomous bioethanol plant, but a transparent decision infrastructure that connects changing plant states with low-carbon operational choices.

## Abbreviations

AI: artificial intelligence
BOD: biochemical oxygen demand
CAPEX: capital expenditure
CBP: consolidated bioprocessing
CCD: central composite design
CHP: combined heat and power
CMMS: computerized maintenance management system
COD: chemical oxygen demand
DCS: distributed control system
DT: digital twin
EFB: empty fruit bunch
EROI: energy return on investment
ETL: extract, transform, load
FER: fossil energy ratio
GHG: greenhouse gas
HEN: heat-exchanger network
HMF: 5-hydroxymethylfurfural
IRR: internal rate of return
KG: knowledge graph
LCA: life-cycle assessment
LCI: life-cycle inventory
LHV: lower heating value
LIMS: laboratory information management system
MESP: minimum ethanol selling price
MTBF: mean time between failures
NER: net energy ratio
NPV: net present value
OPEX: operating expenditure
PAT: process analytical technology
SEC: specific energy consumption
SCADA: supervisory control and data acquisition
SHF: separate hydrolysis and fermentation
SSCF: simultaneous saccharification and cofermentation
SSF: simultaneous saccharification and fermentation
TEA: techno-economic analysis
